# Physical Mechanism of Concrete Damage under Compression

**DOI:** 10.3390/ma12203295

**Published:** 2019-10-11

**Authors:** Hankun Liu, Xiaodan Ren, Shixue Liang, Jie Li

**Affiliations:** 1 Sichuan Institute of Building Research, Chengdu 610081, China; lhksibr@foxmail.com; 2 School of Civil Engineering, Tongji University, 1239 Siping Road, Shanghai 200092, China; lijie@tongji.edu.cn; 3 School of Civil Engineering and Architecture, Zhejiang Sci-Tech University, Hangzhou 310018, China; liangshixue0716@126.com

**Keywords:** concrete, damage, compression, random field, nanoindentation, multiscale

## Abstract

Although considerable effort has been taken regarding concrete damage, the physical mechanism of concrete damage under compression remains unknown. This paper presents, for the first time, the physical reality of the damage of concrete under compression in the view of statistical and probabilistic information (SPI) at the mesoscale. To investigate the mesoscale compressive fracture, the confined force chain buckling model is proposed; using which the mesoscale parameters concerned could be directly from nanoindentation by random field theory. Then, the mesoscale parameters could also be identified from macro-testing using the stochastic damage model. In addition, the link between these two mesoscale parameters could be established by the relative entropy. A good agreement between them from nano- and macro- testing when the constraint factor approaches around 33, indicates that the mesoscale parameters in the stochastic damage model could be verified through the present research. Our results suggest that concrete damage is strongly dependent on the mesoscale random failure, where meso-randomness originates from intrinsic meso-inhomogeneity and meso-fracture arises physically from the buckling of the confined force chain system. The mesoscale random buckling of the confined force chain system above tends to constitute the physical mechanism of concrete damage under compression.

## 1. Introduction

Concrete, a mixture of Portland cement, water, sand and aggregate, hydrated to form cementitious material with micro-crack, void and inhomogeneous [1], exhibits nonlinearity and randomness of mechanical properties. Under loading, concrete and its properties suffer from deterioration, which could be regarded as damage. The concept of damage is firstly developed by Kachanov [2], introduced into concrete material by Dougill soon afterwards [3]. Over decades, concrete nonlinearity, especially the characterization of properties softening, has been modeled by damage mechanics, whose branches include continuum damage mechanics [4,5] and stochastic damage mechanics [6,7]. The former one regards macroscopic homogeneity, however, the latter one pays more attention to mesoscopic inhomogeneity and the progressive transition between different levels. Mazars modeled the degradation of concrete by bi-scalar model [4], based on this, Wu and Li proposed a plastic damage model by introducing the elastoplastic damage release rate [5]. Moreover, to discover the physical mechanism of concrete damage, the latter one, idealized as a parallel fiber bundle including two levels, has been always applied due to its simplicity in reproducing randomness and nonlinearity. Actually, concrete could be always regarded as a set of parallel small concrete rods connected on two ends and deform compatibly. At the macroscale, concrete could be modeled as a fiber bundle, giving a smooth curve reflecting the averaging response. While at the mesoscale, a small concrete rod could be considered as a fiber, exhibiting a kind of elastic-brittle relationship, referring to the previous researches [6,7,8]. Up to now, the fiber bundle model has been used for modeling concrete damage including tensile and compressive damage, under the static loading [7] and the dynamic loading [8]. In addition, Bazant and Pang also systematically investigated the size effect of concrete materials based on the bundle model [9]. It is noted that the physical mechanism for concrete tensile damage could be easily disclosed by the bundle model; however, the physical mechanism for concrete compressive damage remains a mystery.

To disclose the physical mechanism, the physical experiment could always be considered as a direct and effective way. Although it is generally realized that the nonlinearity and randomness of the mechanical properties for concrete are directly related to the fracture of the meso-element and its accumulation, the direct access to the knowledge regarding the local properties has never been provided until the 1970s. Hereafter, Beaudoin and Feldman systematically studied the properties of the autoclaved calcium silicate systems including elastic modulus, micro-strength, micro-hardness and the relationships between the properties by micro-hardness testing, and additionally, suggested the linear relationship between microscale compressive strength and micro-hardness [10]. From Igarashi’s research, the microscale compressive strength and the micro-hardness both increased with increasing curing age and decreasing water to cement ratio, meanwhile, the same relationship as Beaudoin and Feldman was also verified [11]. Zhu and Bartos proposed a novel microindentation method continuously monitoring load and displacement, to assess the elastic modulus and microhardness of the interfacial zone of reinforced concrete [12]. According to the research of Buckle and Durst et al. [13,14], small indentation depths lead to mechanical phase properties, while greater indentation depths result in homogenized material properties. Georgios and Ulm proposed a novel method by means of grid indentation, using deconvolution technique to identify in situ two calcium–silicate–hydrates (C–S–H) phase (low-density C–S–H and high-density C–S–H) [15,16,17]. To anticipate the nature of strength, the dual-nanoindentation technique was proposed to assess the strength of bone and cohesive-frictional materials, suggesting the nanogranular friction responsible for the increased intrinsic resistance in compression [17,18,19]. Vandamme et al. also investigated the nanogranular origin of concrete creep by nanoindentation [20]. Liu et al. combined nanoindentation with random field modeling to study the probabilistic and statistical properties of concrete materials, suggested the multiscale SPI of concrete in a comprehensive manner and proposed a multi-scale random media model for concrete [21]. In addition, Mondal et al. studied the topological structure of cementitious materials using nanoindentation [22]. In short, microhardness testing [10,11], microindentation [12] and the nanoindentation technique [13,14,15,16,17,18,19,20,21,22] appeared successfully, using that which researchers have investigated; the fundamental knowledge including topological structure, mechanical and physical information, SPI, as well as their interrelationships. With the development of material science and technology, the materials genome could be discovered. Through the usage of this, the elementary physical properties and fundamental structural characteristics could also be predicted [23]. Usually, for concrete, a rather complex system, it is crucial and feasible to use the SPI of the intrinsic structure and properties at the nanoscale or mesoscale to investigate macroscopic properties. Unfortunately, the physical mechanism of concrete damage or constitutive relationship under compression remains unknown, despite the great achievements on continuum damage mechanics which fails to reveal the mechanism, and on stochastic damage mechanics (the bundle model, etc.) which has failed to be verified by the physical experiment until nowadays. On the basis of the recent progress on nanoindentation, stochastic mechanics and random media modeling for concrete, the paper focuses on the link between concrete damage and materials SPI at the lower scale, and on the origin of concrete damage.

In this research, the damage of concrete is investigated when subjected to compressive loading. Based on the previous research achievement, it is hypothesized that the fracture of “concrete fiber” originates at the mesoscale. On the one hand, nanoindentation tests are conducted on each constituent of concrete: hardened cement paste (HCP), interfacial transition zone (ITZ) and aggregate. By application of the confined force chain buckling model proposed in this paper, reconstruction technique [21] and random field theory, the SPI of fracture behavior could be obtained for concrete fiber at the mesoscale. On the other hand, the SPI of meso-parameters of concrete could also be recognized from macro-testing employing the stochastic damage model. Thus, this hypothesis could be proven, as long as the SPI of the meso-fiber from the nanoindentation coincides with that recognized from the macro-testing.

## 2. Materials and Methods

### 2.1. Materials and Preparation

**Figure 1:**
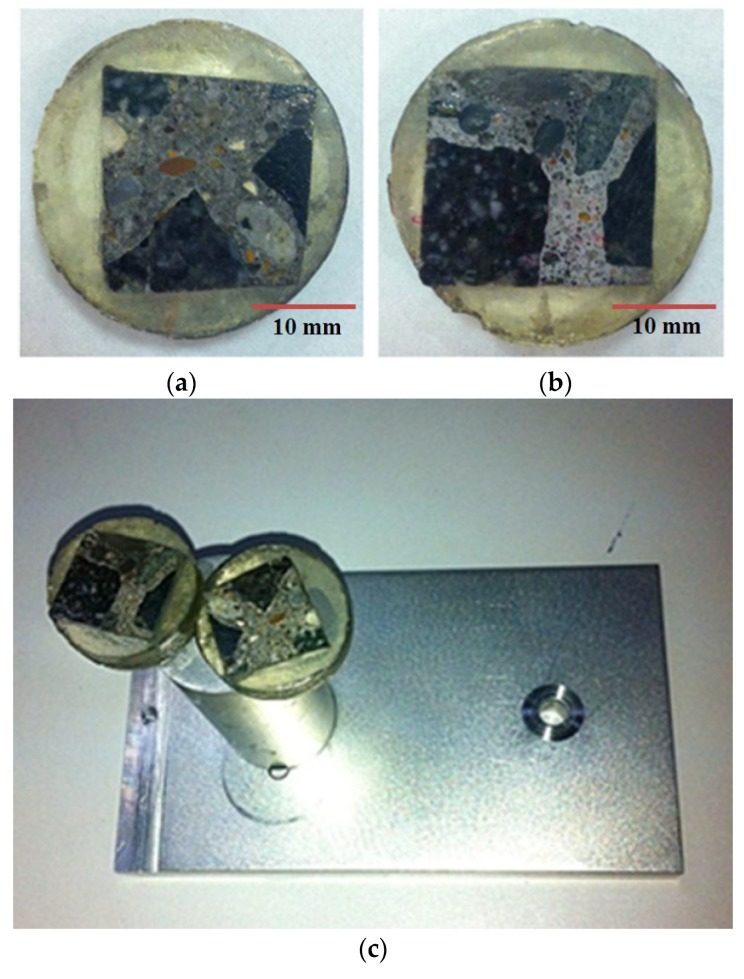
Samples for testing in nanoindenter: (**a**) specimen 1; (**b**) specimen 2; and (**c**) specimens installed.

The material prepared here was ordinary concrete with water: cement: sand: with an aggregate ratio of 0.4:1:2:5. The bars were made measuring 0.1 m × 0.1 m × 0.3 m, and hydrated with the humidity of 95% at the room temperature for three months, which were used for uniaxial compression testing at the macroscale. Then, the prisms were sliced into small specimens with approximate dimensions of 0.02 m × 0.02 m × 0.005 m, which were prepared for nanoindentation. After embedding into the epoxy resin, grinding and polishing with silicon carbide papers, and ultrasonically cleaning, the samples for nanoindentation (Figure 1) were prepared. Details of sample preparation for nanoindentation have been described in the previous study [21].

### 2.2. Nanoindentation

**Figure 2:**
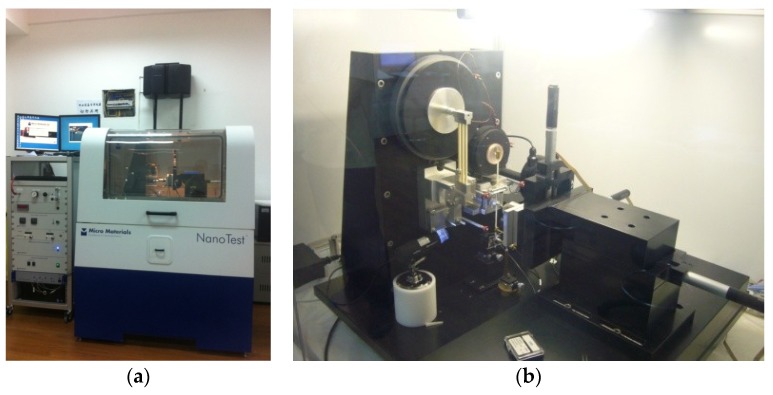
NanoTest Vantage testing system: (**a**) appearance; and (**b**) internal details.

The equipment conducting nanoindentation in the present paper is the NanoTest Vantage system (Figure 2, from Micro Materials Limited in Wrexham, UK) to offer nanomechanical and nanotribological tests, with electromagnetic load application, with a maximum load of 500 mN, load resolution of 3 nN and displacement resolution of 0.002 nN. A series of nanoindentation tests were conducted with a Berkovich tip, with a maximum depth of 300 nm, loading and unloading rate of 0.2 mN/s and holding time of 15 s. According to the approach of Oliver and Pharr [24], indentation hardness *H* and indentation modular *M* were obtained from loading-unloading curves. Furthermore, Young’s modulus linking to the elastic constants of specimen and indenter [25], could be expressed as

(1) $$\frac{1}{M}\overset{}{=}\frac{1-\nu^{2}}{E}+\frac{1-\nu_{i}{}^{2}}{E_{i}}$$

where $E_{i}$ and $\nu_{i}$ are Young’s modulus and Poisson’s ratio of the indenter employed with a given value of 1140 GPa and 0.07; E and ν are that of the tested materials, and the Poisson’s ratio is 0.2. 

Nanoindentation on each constituent of concrete was conducted in the present paper, and the dimensions of the indent lattice for HCP, ITZ and aggregate were all 25 × 20.

### 2.3. Macro-Testing

**Figure 3:**
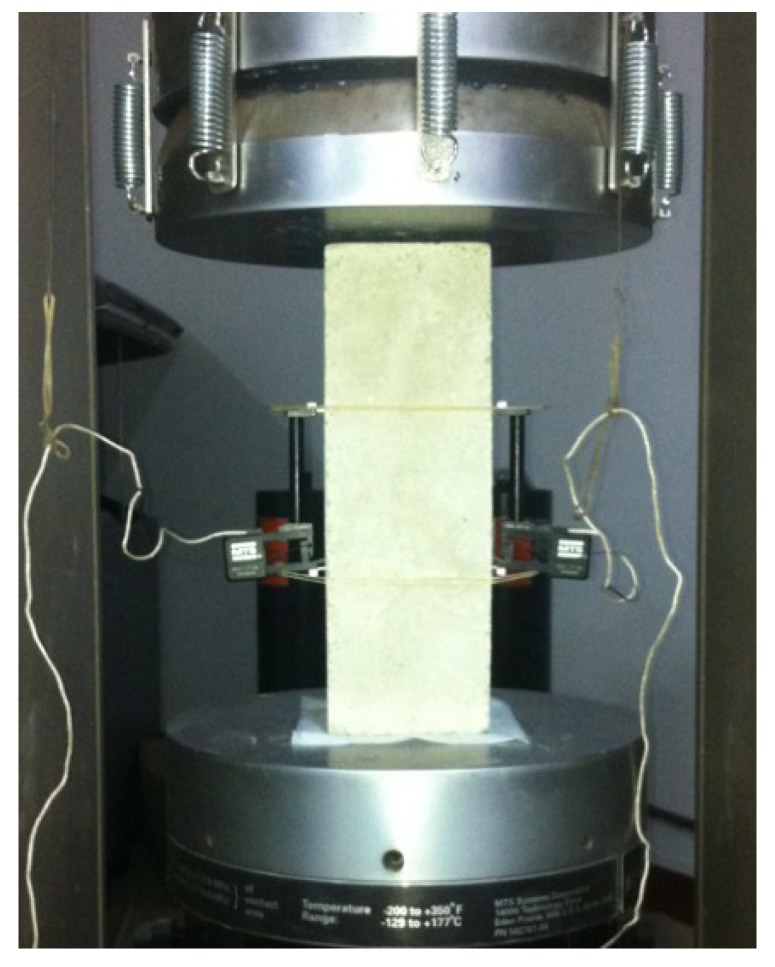
Macro-testing system of uniaxial loading.

A total of seven prism specimens subjected to uniaxial compressive loading were investigated by an electro-hydraulic servo-controlled concrete testing system from MTS Systems Corporation in Eden Prairie, MN, USA (Figure 3). The hinge on the top and the scale marks on the bottom are used to guarantee the axial compression and the accurate centration of the tested samples. The stiffness of 1.1 × 10^10^ N/m is enough to provide the closed-loop controlled compression and the data collection accuracy. A pair of extensometers were installed on opposite sides of the specimen shown in Figure 3, which collected the axial displacement data. And the strain rate was 10^−5^ which guaranteed a static loading.

## 3. Theoretical, Experimental and Numerical Approach

### 3.1. Shear Fracture Strain (SFS) from Nanoindentation

#### 3.1.1. Force Chain Based Modeling for Hardness

**Figure 4:**
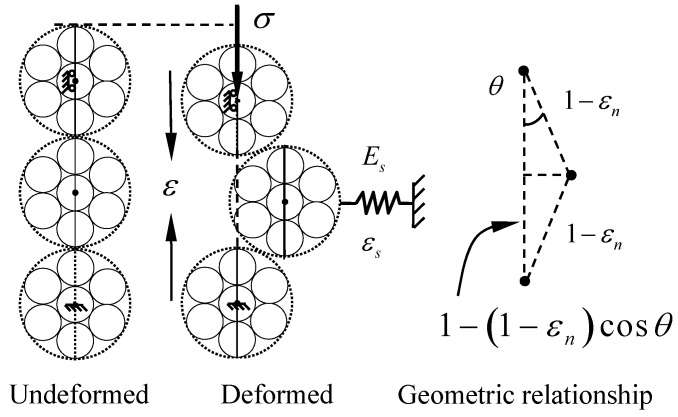
Three-particle force chain model.

Due to the nanogranular nature of C–S–H [26], the aggregative particles of C–S–H could be assumed to yield the confined three-particle force chain buckling mechanism which was firstly proposed by Tordesillas and Muthuswamy [27]. The schematic is shown in Figure 4. Based on the force chain theory, a connection could be established from nanoscale to mesoscale. 

For simplicity and possibility, only the contact force between particles and the lateral supporting force from the surrounding weak force chains are assumed for C–S–H in the present paper. The assumption could be extended to cement paste, ITZ and aggregate, because of the similar characteristics among the constituents of concrete. Next, the contact law is introduced to describe the key features in the force chain model.

##### Contact Models

The homogenized normal stress and tangential stress between particles are respectively

(2) $$\sigma_{n}=E_{n}\varepsilon_{n}^{}$$

(3) $$\sigma_{t}=E_{t}\varepsilon_{t}^{}$$

where $E_{n}$ and $E_{t}$ denote the normal and tangential modulus respectively; $\varepsilon_{n}^{}$ and $\varepsilon_{t}$ are the normal and tangential strain.

To reflect the constraint effect of the weak force chain network, the applied confined pressure could be expressed as follows.

(4) $$\sigma_{s}=E_{s}\varepsilon_{s}^{}$$

where $E_{s}$ and $\varepsilon_{s}^{}$ denote the confined modulus and the corresponding stain.

##### Averaged Potential Energy Density

Considering a certain region (at the mesoscale) of cement paste in the surrounding area of the indenter, when applying compression by a strain ε, the averaged potential energy density $e_{p}$ could be expressed as follows:

(5) $$e_{p}=u-w=\frac{1}{2}E_{n}\varepsilon_{n}^{2}+\frac{1}{2}E_{t}\varepsilon_{t}^{2}+\frac{1}{2}E_{s}\varepsilon_{s}^{2}-\sigma \varepsilon$$

where u is the stored energy density; w is the work density with the stress σ. As shown in Figure 4, the strain $\varepsilon_{n}^{}$, $\varepsilon_{t}$ and $\varepsilon_{s}^{}$ could be rewritten in terms of θ and $\varepsilon_{n}^{}$ as follows:

(6) $$\left|\varepsilon_{t}^{}\right|=\left(1-\varepsilon_{n}^{}\right)\sin\theta \approx \sin\theta$$

(7) $$\varepsilon_{s}^{}=\left(1-\varepsilon_{n}^{}\right)\sin\theta \approx \sin\theta$$

(8) $$\varepsilon =1-\left(1-\varepsilon_{n}\right)\cos\theta$$

Substituting Equations (6)–(8) into Equation (5), one obtains the potential energy density:

(9) $$e_{p}=\frac{1}{2}E_{n}{\left(\varepsilon_{n}\right)}^{2}+\frac{1}{2}E_{t}{\left(\sin\theta \right)}^{2}+\frac{1}{2}E_{s}{\left(\sin\theta \right)}^{2}-\sigma \left[1-\left(1-\varepsilon_{n}\right)\cos\theta \right]$$

It is evidently observed that the normal strain and the tangential strain could be decoupled.

##### Critical Load

According to the principle of resident potential energy, the partial derivative of the averaged potential energy density with respect to each degree of freedom should be zero, we get

(10) $$\frac{\partial e_{p}}{\partial \theta }=0,\;\frac{\partial e_{p}}{\partial \varepsilon_{n}}=0$$

Substituting Equation (9) into Equation (10), and solving Equation (10), the relationship between the stress and the normal strain yields Equation (11).

(11) $$\sigma =\frac{\left(E_{t}+E_{s}\right)\cos\theta }{1-\varepsilon_{n}},\;\varepsilon_{n}=\frac{\sigma \cos\theta }{E_{n}}$$

From Equation (11), an apparently stable path for the confined force chain could be achieved when the force chain structure keeps straight from the beginning of loading, which corresponds to

(12) $$\sigma =E_{n}\varepsilon_{n}$$

Solving Equation (11), σ, $\varepsilon_{n}$ and ε could be obtained, which yields

(13) $$\sigma =\frac{E_{n}}{2\cos\theta }\left(1-\sqrt{1-4\cos\theta \frac{\left(E_{t}+E_{s}\right)}{E_{n}}}\right)$$

(14) $$\varepsilon_{n}=\frac{1}{2}\left(1-\sqrt{1-4\cos\theta \frac{\left(E_{t}+E_{s}\right)}{E_{n}}}\right)$$

(15) $$\varepsilon =1-\frac{1}{2}\left(1+\sqrt{1-4\cos\theta \frac{\left(E_{t}+E_{s}\right)}{E_{n}}}\right)\cos\theta$$

Another stable path could be acquired when θ is zero and this critical point with the stress and strain of Equations (16) and (17) corresponds to a buckling load of the force chain system.

(16) $$\sigma_{cr}=\frac{E_{n}}{2}\left(1-\sqrt{1-4\frac{\left(E_{t}+E_{s}\right)}{E_{n}}}\right)$$

(17) $$\varepsilon_{cr}=\varepsilon_{n}=\frac{1}{2}\left(1-\sqrt{1-4\frac{\left(E_{t}+E_{s}\right)}{E_{n}}}\right)$$

To conduct Taylor expansion for Equation (16) and Equation (17) at $E_{t}+E_{s}/E_{n}=0$, the results obtained are as follows.

(18) $$\varepsilon_{cr}=\frac{1}{2}\left(1-\sqrt{1-4\frac{\left(E_{t}+E_{s}\right)}{E_{n}}}\right)\approx \frac{E_{t}+E_{s}}{E_{n}}$$

(19) $$\sigma_{cr}=E_{n}\varepsilon_{cr}\approx E_{t}+E_{s}$$

As easily seen from Equation (19), a mixed-mode of shearing ($E_{t}$) and constraint ($E_{s}$) is included in the critical load, which means that the critical compressive load of the meso-element is attributed to the meso-element itself and the surrounding materials. With the framework of hardness theory, the hardness value H is the pressure at a limiting condition where the pressure keeps constant with increasing load [28]. Meanwhile, for indentation on cement paste, there is also a mixed fracture mode by cutting and hydrostatic pressure under the indenter at the limiting state [11]. Based on the statement above, the indentation hardness could be equivalent to the constrained compressive strength $\sigma_{c}$ of the meso-element confined by the surrounding material around the indenter:

(20) $$\sigma_{c}=\sigma_{cr}\approx E_{t}+E_{s}=H$$

#### 3.1.2. Constrained SFS

Over decades, researchers have investigated the relationship between the strength and the micro-hardness, with the conclusion that for cementitious materials (frictional materials), the hardness and the yield stress relationship of the form *H*/*Y* were reported on the order of 20–30 [29]. Similarly, in Ref. [11], the ratio was found to be 2.7–3 more order of magnitude than that of metals, which was in the range of 30–60. As known from early on, the ratio of metal discussed above, named “the constraint factor”, was evidently less than that of cementitious materials, due to the frictional effect on the hardness within the cohesive-frictional materials.

Based on the previous researches [11,29] and Equation (20), the relationship between the hardness H and the compressive strength $\sigma_{y}$ was followed by introducing a constraint factor C:

(21) $$C=\frac{H}{\sigma_{y}}$$

With the assumption of elastic-brittle property of mesoscale element aforementioned [6,7,8], the SFS $\Delta_{1,s}\left(x\right)$ from nanoindentation could be rewritten as

(22) $$\Delta_{1,s}\left(x\right)=\frac{\sigma_{y}}{E}=\frac{H}{CE}=\frac{\Delta_{con}}{C}$$

where $\Delta_{\mathrm{con}}$ is defined as the constrained SFS. Combining Equation (1) and Equation (22), the constrained SFS could be rewritten as

(23) $$\Delta_{con}=\frac{H}{E}=\frac{H}{1-\nu^{2}}\left(\frac{1}{M}-\frac{1-\nu_{i}{}^{2}}{E_{i}}\right)$$

#### 3.1.3. Random Field Modeling and Statistical Modeling

Due to the attribute of a sufficient large degree of disorder, the knowledge of the probabilistic characteristics of concrete is fundamental to understanding the intrinsic random microstructure, even the nanostructure. Generally, random field theory deals effectively with the complex distributed disordered system [30]. As the random heterogeneous materials, Torquato has made considerable progress on 2D and 3D microstructure characterization, also on the relationship between mechanical properties and microstructure [31]. However, it is very difficult to use 2D or 3D modeling to investigate the physical mechanism of concrete damage, due to more parameters and complex simulation execution. To make it convenient and effective, the 1D random field is still adopted in the present paper.

The random field could be defined as homogeneous, as long as the mean $m_{X}\left(t_{j}\right)$ and the covariance $R_{X}\left(t_{j},t_{j}+\tau \right)$ as follows keep constant with the variance of space, in other words, these values only depend on the relative distance τ.

(24) $$m_{X}\left(t_{j}\right)=E\left[X\left(t_{j}\right)\right]$$

(25) $$R_{X}\left(t_{j},t_{j}+\tau \right)=E\left[X\left(t_{j}\right)X\left(t_{j}+\tau \right)\right]$$

where E[ ] is the expected operator, $X\left(t_{j}\right)$ is the observation on the random series with respect to $t_{j}$.

To investigate the statistical characteristics, Kolmogorov–Smirnov test (referred to as the K–S test) could be adopted to acquire the probabilistic density function (PDF) of each point of the random series (containing six sections). To execute the K-S test, the main procedure is outlined as follows:

(1) Choose a sample $X_{i}$ from the population X and rearrange sample values $x_{i}$ in increasing order of magnitude.
(2) Compute the observed cumulative distribution function (CDF) $F_{n}\left(x_{i}\right)$ at each ordinal sample value.
(3) Estimate the parameters of the hypothesized distribution as described below based on the observed data and determine the theoretical CDF $F\left(x_{i}\right)$ at the same sample value above using the hypothesized distribution.
(4) Form the differences $\left|F_{n}\left(x_{i}\right)-F\left(x_{i}\right)\right|$, and calculate the statistics:
  (26) $$D=\overset{n}{\underset{i=1}{\max}}\left\{\left|F_{n}\left(x_{i}\right)-F\left(x_{i}\right)\right|\right\}$$
(5) Select a value of α and determine the critical value $D_{\alpha }$.
(6) Accept or reject the testing hypothesis by comparing D and $D_{\alpha }$.

The procedure stated above is the classic K-S test process. However, since the critical value is approximate, the null hypothesis is usually rejected or accepted by comparing the returned P value and the significance level α.

In this study, the hypothesized PDF commonly used in civil engineering could be made including normal distribution, lognormal distribution, Weibull distribution and gamma distribution. Then, the estimated PDF could be acquired by executing the K-S test. According to Ref. [21], there is only a little difference between PDFs using the mean estimated parameters of six sections and the parameters given by a maximum possibility criterion proposed by the authors. Therefore, in this paper, the PDF with the mean estimated parameters of six points could be referred to as the best estimate.

By conducting the probabilistic and statistical modeling on the constrained SFS, the 1D PDF of constrained SFS for each constituent of concrete could be obtained.

### 3.2. SFS from the Macro-Testing

**Figure 5:**
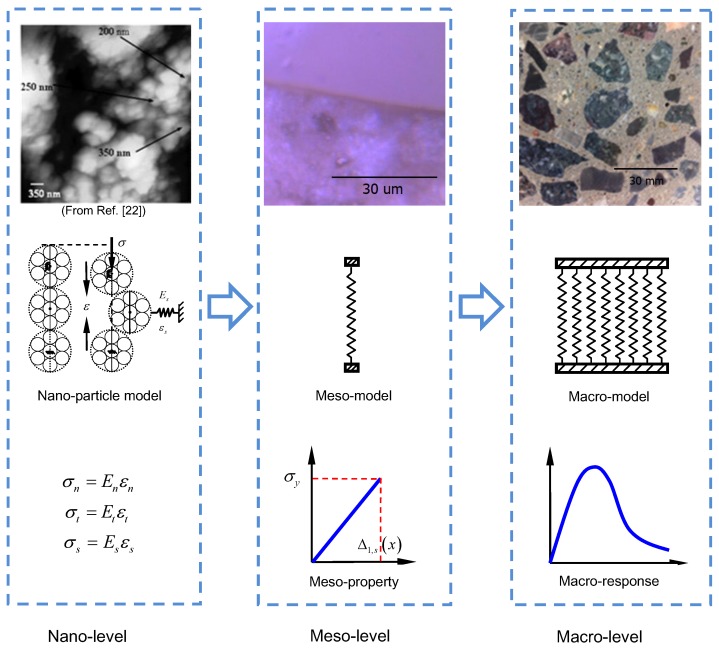
Concrete modeling at the nano-, meso- and macro-scales.

Generally, complex global behaviors could be captured on the basis of the fiber bundle model whose individual element is endowed with a simple response (elastic-brittle prosperity shown in Figure 5). Under compressive loading, one of these fibers would fail when the overall strain exceeds the random SFS denoted by the random variable $\Delta_{2,s}\left(x\right)$, which could be considered to be a homogenous lognormal random field with the mean λ and the standard deviation ζ in Refs. [6,7,8]. According to Refs. [7,8], the damage of the fiber bundle represented by d(ε) could be defined as follows

(27) $$d\left(\varepsilon \right)=\int_{0}^{1}H\left[\varepsilon -\Delta_{2,s}\left(x\right)\right]dx$$

where H[⋅] is the Heaviside function, ε is the elastic strain, $\Delta_{2,s}\left(x\right)$ is the 1D random field for fracture strain, and x is the spatial coordinate of the meso-fiber. In the present work, the focus would be on the expected value of the damage variable d(ε) given as

(28) E[d]=F(ε)

where F(ε) denotes the first-order cumulative distribution function of $\Delta_{2,s}\left(x\right)$.

The 1D expected stress and strain relationship could be expressed as follows

(29) $$E\left[\sigma \right]=\left[1-F\left(\varepsilon \right)\right]E_{0}\varepsilon$$

where σ denotes the stress, and $E_{0}$ denotes the initial elastic modulus.

### 3.3. Multiscale Approach

Figure 5 shows the topological structure, physical model and mechanical properties at three scales. At the nanoscale, a confined force chain buckling model was established for force-chain based materials, which could be extended to the mesoscale using the principle of resident potential energy, to investigate the relationship between the compressive strength and the hardness for the mesoscale concrete element. Meanwhile, at the mesoscale, the mesoscale parameters for concrete damage under compression could be not only from the identification combining model results with macro testing results but also from the direct experiment (nanoindentation). Then, further than that, the former one could be regarded as a traditional method: the parameters at the lower scale could be recognized provided that a reasonable model and the macroscale experiment results are given. The latter pays more attention to the verifiability of the model and the development of the microscale testing techniques. Actually, the fiber bundle model would be an optimal and convenient model to connect these three scales.

On the one hand, each concrete constituent (HCP, ITZ and aggregate) could be modeled as a random field using the observed values from nanoindentation tests. Performing “maximum possibility criterion” [21], the knowledge of PDF for constrained SFS $\Delta_{\mathrm{con}}$ could be provided, with which SFS $\Delta_{1,s}\left(x\right)$ could also be obtained using Equation (22) (in Section 3.1). Then, employing stochastic damage theory, the statistical characteristics of SFS $\Delta_{2,s}\left(x\right)$ could also be obtained including the mean and the standard deviation of a homogenous lognormal random field, by comparing the macroscale experimental stress and strain curves with the model results (in Section 3.2). Finally, the constraint factor in Equation (22) could be recognized by comparing $\Delta_{1,s}\left(x\right)$ with $\Delta_{2,s}\left(x\right)$ through the relative entropy (in Section 4.3).

## 4. Results and Discussion

### 4.1. Concrete Damage SPI from Nanoindentation

Specifically, to investigate the SPI of concrete damage, the phase SPI could be generated firstly due to the obvious bound among each constituent of concrete. Combined with the phase SPI, concrete SPI could be reproduced by the reproduction technique [21]. 

#### 4.1.1. Phase SPI and Random Field Theory

**Figure 6:**
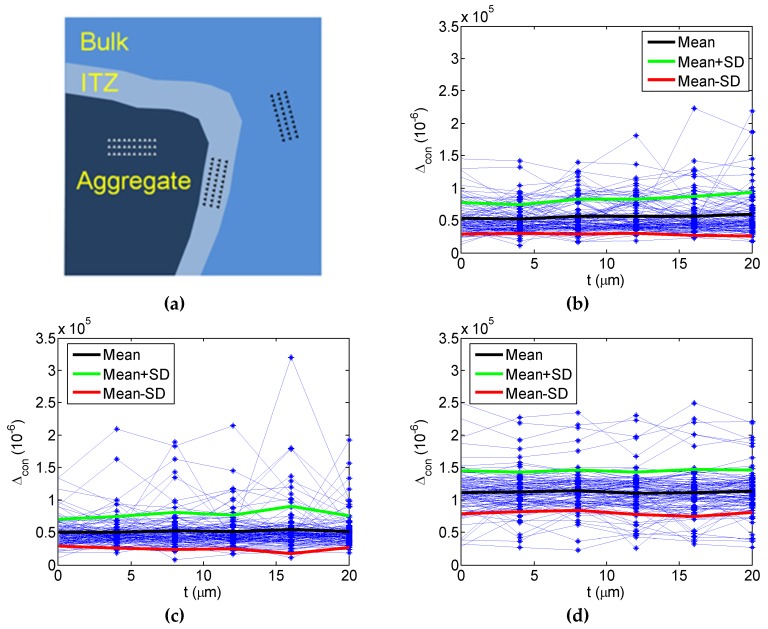
1D random filed observations: (**a**) the indent zones; the samples, the mean and the standard deviation for the constrained SFS of (**b**) HCP, (**c**) ITZ and (**d**) aggregate.

To characterize the probability distribution of properties from nanoindentation, three zones including HCP, ITZ and aggregate were selected randomly shown in Figure 6a before nanoindentation. Following random field modeling of nanoindentation results, it leads to a sample number of 100 for HCP, aggregate and ITZ, respectively, with respect to the constrained SFS $\Delta_{\mathrm{con}}$. In Figure 6b–d, they show the samples of the random field, and the corresponding mean and standard deviation for HCP, ITZ and aggregate, respectively. From the first and second-order statistical characteristics, the mean value and the standard deviation calculated both keep in constant. It is clear that the 1D random filed for the concrete constituent herein is belonging to a stationary random process. Notably, concrete constituents could be considered to be homogeneous from the probabilistic and statistical standpoint, although concrete is well-known as a kind of inhomogeneous material. Actually, concrete could also be regarded as homogenous, provided that one considers the complex materials as random media.

**Figure 7:**
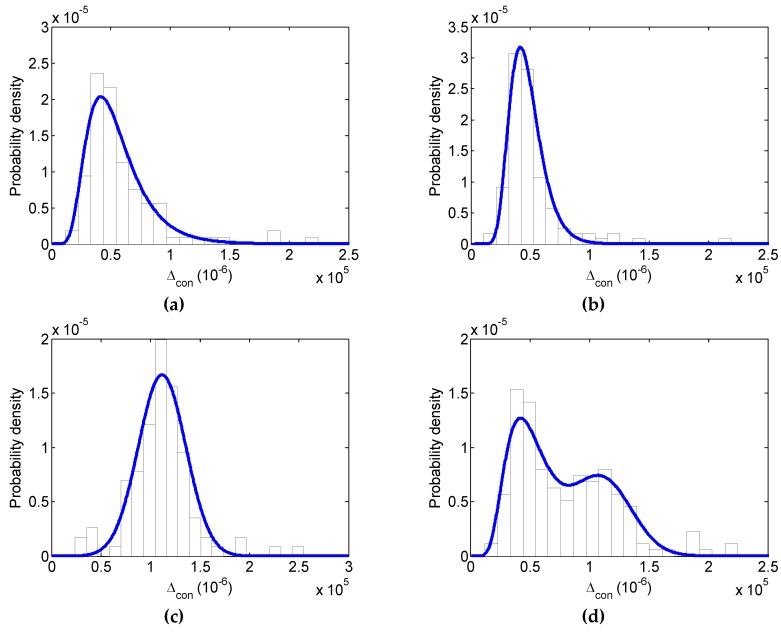
Reconstruction of the constrained SFS for concrete: histograms and theoretical probability density curves for (**a**) HCP, (**b**) ITZ, (**c**) aggregate, and (**d**) concrete reconstructed with Equation (30).

By performing “maximum possibility criterion” [21], the 1D PDF for the random field could be obtained. It shows the optimal parameters in Table 1 for the 1D PDF of the random field by the simplex method [32,33]. Figure 7a–c gives the histograms of the constrained SFS for each concrete constituent together with the theoretical PDF using the optimal parameters in Table 1. The comparisons between the histograms and the theoretical PDFs show better agreement.

#### 4.1.2. Synthesis Technique of Concrete SPI

As mentioned above, each constituent of concrete materials exhibits randomness, which constitutes the complex, distributed disordered system. The PDF of concrete, regarded as a random media [21], could be directly obtained by adding the individual PDF, expressed as follows

(30) $$\phi \left(x\right)=\sum_{i=1}^{n}f_{i}\phi_{i}\left(x\right)\;i=1,2,3$$

where x denotes the property of concrete or its constituent; $f_{i}$ denotes the volume fraction of HCP, ITZ or aggregate; $\phi_{i}\left(x\right)$ denotes the PDF of the concrete constituent; ϕ(x) denotes the reconstructed concrete PDF. According to the previously reported result [21], a volume fraction ratio of HCP: aggregate: ITZ is around 0.611:0.387:0.002, and the corresponding random media distributions of concrete are displayed in Figure 7d. It is evident that the histograms and the theoretical PDFs of the constrained SFS are in close agreement with each other.

One may argue that the optimal distributions listed in Table 1 remain subjective. However, the theoretical distribution obtained could really model the primary characteristics for each constituent, even concrete. In the meantime, it is really a choice for engineers and researchers to statistically and probabilistically model concrete from a practical point of view. Especially, the distribution of concrete shown in Figure 7d provides the physical reality for concrete damage under compression in Section 4.2.

### 4.2. Concrete Damage SPI from Macro-testing

**Figure 8:**
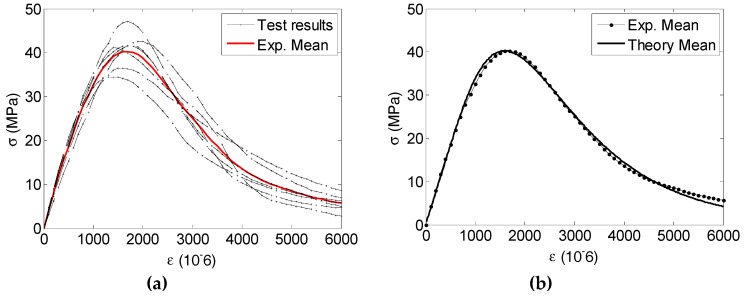
Stress-strain curves under uniaxial compression: (**a**) the stress-strain curves of seven prisms and a mean stress vs. strain curve; and (**b**) comparison of expected experimental and theoretical results with the best-fit parameters.

**Figure 9:**
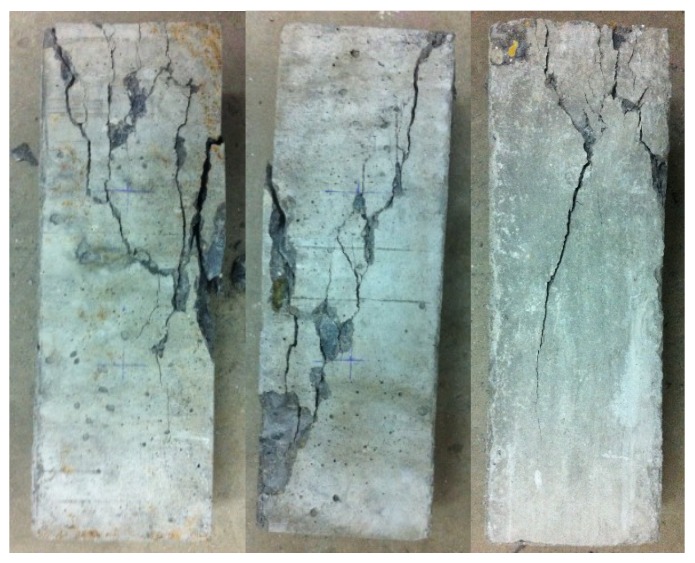
Photos of failed specimens.

To validate the PDF of SFS from nanoindentation, the behaviors of the total seven prism specimens measuring 0.1 m × 0.1 m × 0.3 m subjected to uniaxial compressive loading were investigated by MTS in this research. Figure 8a shows the uniaxial test results of the concrete specimens at the macroscale. It is observed from Figure 8a that all the stress-strain responses are displayed, and they appear randomly. Taking expectation of the stress with respect to the strain, the experimental mean stress and strain relationship could be also plotted. By comparing the theoretical mean stress-strain result from Equation (29) with the experimental result, the mesoscale parameters are found to be *E* = 37.00 GPa, *λ* = 7.62 and *ξ* = 0.52. The theoretical and experimental results are displayed in Figure 8b, which indicates that the theoretical result agrees well with the experimental one. From the failure patterns, the classic failure pattern with a major diagonal crack could be observed shown in Figure 9.

In the present paper, the focus would be on the mean stress and strain relationship, from which the parameters including the mean value and the standard deviation of the distribution could be recognized. However, the standard deviation stress and strain relationship could also be plotted in the figure, which would lead to the relative length of the random field for concrete materials. That is also a key point deserving further research.

### 4.3. Constraint Factor: Linking $\Delta_{1,s}$ with $\Delta_{2,s}$

For establishing the link between SFS $\Delta_{1,s}$ (with an unknown constraint factor C) and $\Delta_{2,s}$, the relative entropy theory could be employed to study on the similarity between these two distributions of $\Delta_{1,s}$ and $\Delta_{2,s}$. In statistics, the relative entropy could be regarded as a measure of the distinguishability between two probability density distributions, which is also called Kullback–Leibler divergence [34]. For the probability distributions of two discrete random variables P and Q, the relative entropy $D_{\mathrm{KL}}\left(P\|Q\right)$ could be expressed as

(31) $$D_{\mathrm{KL}}\left(P\|Q\right)=\sum_{i}P\left(i\right)\ln\frac{P\left(i\right)}{Q\left(i\right)}$$

Assume that the random field in the stochastic damage model following a lognormal distribution is represented by *P*, and that from the nanoindentation by *Q*. Apparently, a distinct relative entropy $D_{\mathrm{KL}}\left(P\|Q\right)$ would be calculated with different constraint factors, the smallest one of which means a minimum difference between *P* and *Q*. In other words, the constraint factor C meeting the minimum $D_{\mathrm{KL}}\left(P\|Q\right)$ is an optimal value for concrete.

**Figure 10:**
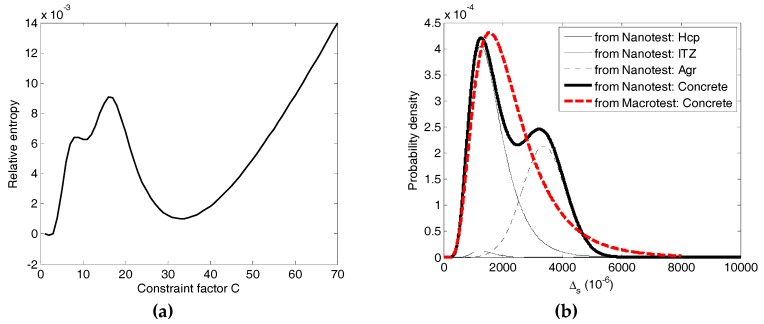
SFS from the nano- and macro-testing: (**a**) the relative entropy vs. the constraint factor. (**b**) PDFs from two different scales corresponding to the smallest relative entropy.

Figure 10a shows the relative entropy with different constraint factors. It is clear that when the constraint factor is very small, the result of Equation (31) would approach zero. In other words, the constraint factor less than five is not of any meaning for concrete materials, despite the pseudo smaller relative entropy. While the relative entropy corresponding to the constraint factor C=33.12 approaches the minimum, giving the knowledge that the discrepancy between these two PDFs is the minimum, namely, they are in good agreement. Moreover, the constraint factor identified agrees well with the previously reported results [11,24]. Figure 10b shows the detailed PDFs of concrete constituent together with the PDFs of $\Delta_{1,s}$ and $\Delta_{2,s}$ obtained above. It is easy to see that the distribution recognized from the macro-test is an ideal lognormal distribution, while the distribution reconstructed from the nano-test is a two-peak distribution, where the first peak is attributed to HCP and the second one is ascribed to the aggregate. It is interesting to note that the difference between probability density at around ∆ = 3400 (the second peak) reaches a larger value, which is attributed to HCP and ITZ playing a more important role than the aggregate during damage evolution. From the failure patterns (seeing Figure 9), through the main crack it is also observed that a small amount of coarse aggregate was broken apart.

From the nanoscale to mesoscale, SFS could be generated based on the nanoindentation, which shows that the meso-fracture arises physically from the bulking of confined force chain system at the mesoscale. From the mesoscale to macroscale, SFS could be recognized based on the macro-testing, which provides the information that the damage of concrete is strongly dependent on the random fracture of mesoscale concrete element. In a word, the concrete damage under compression results from the random buckling of confined force chain system at the mesoscale.

## 5. Conclusions

In summary, this study sets a framework to investigate the damage of concrete under compression based on the SPI. Base on the multiscale research including experimental results, theoretical derivation and numerical analysis, the following conclusions could be drawn:

(1) The confined force chain buckling model proposed indicates the relationship between mesoscale strength and mesoscale hardness. The indentation hardness could be equivalent to the constrained compressive strength confined by the surrounding material around the indenter. The nanoindentation combined with the proposed model and random field theory provides direct access to the SPI of mesoscale fracture behavior of concrete. Meanwhile, the mesoscale fracture behavior of each constituent follows the homogeneous random field.
(2) Nanoindentation combined with macro-testing under compression could lead to the constraint factor linking two scales from mesoscale to macroscale, by comparing the difference between distributions of mesoscale fracture behavior from nano- and macro-testing. This multiscale method provides an effective way to investigate concrete damage under compression, to offer the physical reality of concrete damage evolution, and to estimate the effect of concrete constituents on damage evolution. Up to now, it is interesting to see that the nature of mechanical properties, e.g., strength, creep and damage, could be anticipated based on the SPI by using nanoindentation [17,18,19,20].
(3) At the mesoscale, the meso-fracture arises physically from the bulking of the confined force chain system. At the macroscale, the concrete damage is strongly dependent on the random fracture at the mesoscale. From mesoscale to macroscale, the accumulation of mesoscopic fracture results in the macroscopic damage. Notably, the mesoscale inhomogeneity and the mesoscale confined bulking are intrinsic to concrete, which may constitute the physical mechanism controlling concrete damage subjected to compression.
(4) Our investigation provides the possibility to control damage and to strengthen cementitious materials. Additionally, evaluating the macroscopic properties based on the SPI at the lower scales could be a feasible option. However, in the present paper, only one concrete mix was studied. The concrete materials with higher and smaller water to cement ratios still deserve further investigation and thus the effect of concrete constituents, especially the aggregate on damage evolution could be systematically investigated in the future. Reasonable speculation could be given that the greater the concrete strength, the smaller difference in the second peak in Figure 10b, and vice versa.

## Figures and Tables

**Table 1:** Optimal parameters for the 1D PDF.

Constituents	Properties	Distribution Type	Optimal Parameters	
			Mean Value	Standard Deviation
HCP	Δ_con_	Lognormal distribution	10.82	0.43
ITZ	Δ_con_	Lognormal distribution	10.72	0.29
Agr	Δ_con_	Normal distribution	111730	23910

## References

[1] Breysse D.J. (1990). Probabilistic formulation of damage-evolution law of cementitious composites. J. Eng. Mech..

[2] Kachanov L.M. (1999). Rupture time under creep conditions. Int. J. Fract..

[3] Dougill J.W. (1976). On stable progressively fracturing solids. Angew. Math. Phys..

[4] Mazars J. (1986). A description of micro- and macroscale damage of concrete structures. Eng. Fract. Mech..

[5] Wu J.Y., Li J., Rui F. (2006). An energy release rate-based plastic-damage model for concrete. Int. J. Solids. Struct..

[6] Kandarpa S., Kirkner D.J. (1996). Stochastic damage model for brittle materiel subjected to monotonic loading. J. Eng. Mech..

[7] Li J., Ren X.D. (2009). Stochastic damage model of concrete based on energy equivalent strain. Int. J. Solids. Struct..

[8] Ren X.D., Li J. (2013). A unified dynamic model for concrete considering viscoplasticity and rate-dependent damage. Int. J. Damage. Mech..

[9] Bazant Z.P., Pang S.D. (2007). Activation energy based extreme value statistics and size effect in brittle and quasibrittle fracture. J. Mech. Phys. Solids.

[10] Feldman R.F., Huang C.Y. (1985). Properties of Portland cement-silica fume pastes II. Mechanical properties. Cem. Concr. Res..

[11] Igarashi S., Bentur A., Mindess S. (1996). Characterization of the microstructure and strength of cement paste by microhardness testing. Adv. Cem. Res..

[12] Zhu W., Bartos P.J.M. (1997). Assessment of interfacial microstructure and bond properties in aged GRC using a novel microindentation method. Cem. Concr. Res..

[13] Conrad H., Westbrook J.W. (1973). The Science of Hardness Testing and Its Research Applications.

[14] Durst K., Goken M., Vehoff H. (2004). Finite element study for nanoindentation measurements on two-phase materials. J. Mater. Res..

[15] Constantinides G., Ulm F.J., van Vliet K.J. (2003). On the use of nanoindentation for cementitious materials. Mater. Struct. Rilem..

[16] Dejong M.J., Ulm F.J. (2007). The nanogranular behavior of C–S–H at elevated temperatures (up to 700 °C). Cem. Concr. Res..

[17] Ulm F.J., Vandamme M., Bobko C., Ortega J.A. (2010). Statistical indentation techniques for hydrated nanocomposites: concrete, bone, and shale. J. Am. Ceram. Soc..

[18] Tai K., Ulm F.J., Ortiz C. (2006). Nanogranular origins of the strength of bone. Nano Letters.

[19] Ganneau F.P., Constantinides G., Ulm F.J. (2006). Dual-indentation technique for the assessment of strength properties of cohesive-frictional materials. Int. J. Solids. Struct..

[20] Vandamme M., Ulm F.J., Bazant Z.P. (2009). Nanogranular Origin of Concrete Creep. PNAS USA..

[21] Liu H.K., Ren X.D., Li J. (2018). Indentation tests based multi-scale random media modeling of concrete. Constr. Build. Mater..

[22] Mondal P., Shah S.P., Marks L.D. (2008). Nanoscale characterization of cementitious materials. ACI Mater. J..

[23] Alkhateb H., Al-Ostaz A., Cheng A.H.D., Li X.B. (2013). Materials Genome for Graphene-Cement Nanocomposites. J. Nanomech. Micromech..

[24] Oliver W.C., Pharr G.M. (1992). An improved technique for determining hardness and elastic modulus using load and displacement sensing indentation experiments. J. Mater. Res..

[25] Pharr G.M., Oliver W.C., Brotzen F.R. (1992). On the generality of the relationship between contact stiffness, contact area, and elastic modulus during indentation. J. Mater. Res..

[26] Constantinides G., Ulm F.J. (2007). The nanogranular nature of C–S–H. J. Mech. Phys. Solids.

[27] Tordesillas A., Muthuswamy M. (2009). On the modeling of confined buckling of force chains. J. Mech. Phys. Solids.

[28] Fischer-Cripps A.C. (2011). Nanoindentation.

[29] Kholmyansky M., Kogan E., Kovler K. (1994). On the hardness determination of fine-grained concrete. Mater. Struct..

[30] Vanmarcke E. (2010). Random Fields: Analysis and Synthesis.

[31] Torquato S. (2002). Random Heterogeneous Materials: Microstructure and Macroscopic Properties.

[32] Nelder J.A., Mead R. (1965). A Simplex Method for Function Minimization. Comput. J..

[33] Mathews J.H., Fink K.D. (1999). Numerical Methods Using MATLAB.

[34] Sobezyk K., Trebicki J. (1990). Maximum entropy principle in stochastic dynamics. Probabilist. Eng. Mech..

